# Omnidirectional Interaction Networks Modulating Phenotypic Plasticity in the Gut Microbiota Towards Ulcerative Colitis

**DOI:** 10.1002/advs.78043

**Published:** 2026-10-06

**Authors:** Huizhen Li, Jiayi Li, Ang Dong, Xiaoyan Guo, Qiuyue Tu, Zelin Feng, Haojia Lin, Yanru Zhang, Yuxuan Hao, Huiying Gong, Yihan Meng, Yu Wang, Shing‐Tung Yau, Xiaocang Cao, Rongling Wu, Xinjuan Liu

**Affiliations:** ^1^ Department of Gastroenterology and Hepatology Tianjin Medical University General Hospital Tianjin China; ^2^ Department of Gastroenterology Integrative Microecology Clinical Center Shenzhen Key Laboratory of Gastrointestinal Microbiota and Disease Shenzhen Clinical Research Center for Digestive Disease Shenzhen Technology Research Center of Gut Microbiota Transplantation Shenzhen Hospital of Southern Medical University Shenzhen China; ^3^ Department of Gastroenterology Beijing Chaoyang Hospital Capital Medical University Beijing China; ^4^ Beijing Key Laboratory of Topological Statistics and Applications for Complex Systems Beijing Institute of Mathematical Sciences and Applications Beijing China; ^5^ Bellevue High School Bellevue Washington USA; ^6^ Institute of Statistics and Big Data Renmin University of China Beijing China; ^7^ Yau Mathematical Sciences Center Tsinghua University Beijing China

**Keywords:** evolutionary game theory, gut microbiota, idopNetwork, phenotypic plasticity, ulcerative colitis

## Abstract

Changes to gut microbiomes are linked to inflammatory bowel disease, but little is known about how microbial change is modulated in complex gut microbiota systems. Here, we address this issue by revealing the influence of microbial interactions on gut microbiota response to ulcerative colitis (UC) using a spouse‐pair cohort design composed of an UC patient and his healthy spouse living in the same household environment. We calculate the difference in abundance of a microbial species between each pair of spouses as a proxy of UC‐induced phenotypic plasticity (uPP). We classify all microbes into functionally distinct modules based on the similarity of uPP across household environments. An integrative network and GLMY homology analysis identifies a pathobiont‐centric hub module and its disease‐specific topological motifs, characterized by UC‐associated microbial dysbiosis. We find that a marked increase in microbial plasticity is mediated by profound restructuring of microbial interactomes in the microbial communities. By inferring multilayer interactome networks, our model positions phenotypic plasticity as a systematic mechanistic force of UC, paving the way for its ecology‐informed diagnostics and interventions.

## Graphical abstract

This study employs a spouse‐pair cohort design and an idopNetwork model that integrates evolutionary game theory, predator‐prey dynamics, and developmental modularity theory to reconstruct maximally informative microbial interaction networks. This approach reveals how interspecies interactions drive the phenotypic plasticity of the gut microbiota in ulcerative colitis, thereby elucidating the mechanistic basis of UC‐associated dysbiosis.

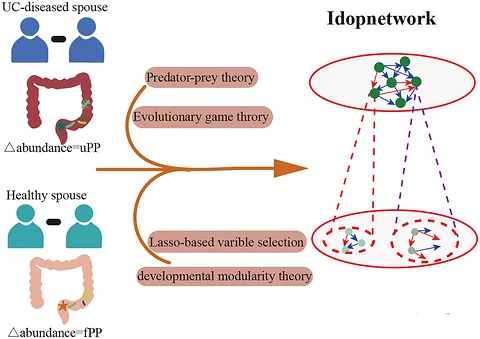

## 1 Introduction

The gut microbiota is regarded as the second genome of the human body that exerts remarkable effects on the host's metabolism, immune function, and pathogen defense and, therefore, overall health [1, 2, 3]. Previous studies have shown that changes in human health from a healthy state to a disease state accompany changes in the composition and diversity of the gut microbiota [4, 5, 6]. From an ecological perspective, the change of a microbe in response to the change of health condition is called phenotypic plasticity, a widespread phenomenon that reflects the capacity of organisms to adapt to environmental change [7, 8, 9, 10, 11, 12]. While phenotypic plasticity is thought to represent a developmental mechanism for evolution [13], it is also regarded as a quantitative trait subject to evolution [14]. Both of these views about phenotypic plasticity suggest that it is a biological process driving evolution and speciation under natural selection [15, 16, 17, 18].

While the concept of phenotypic plasticity can be leveraged to interpret how the gut microbiota ecologically shifts from symbiosis to dysbiosis, no study has been initiated to associate microbial plasticity with the change of health state and unravel the mechanisms underlying this association. In this study, we design a family‐based cohort to investigate the mechanistic role of gut microbiota plasticity in ulcerative colitis (UC), a chronic recurring inflammatory bowel disease [19, 20]. This cohort is composed of UC patients and their healthy spouses living in the same household environment. Thus, the difference in the abundance of a microbial species between UC‐diseased and UC‐free couples from the same family represents a proxy for UC‐induced microbial phenotypic plasticity of this species, reflecting its eco‐evolutionary capacity to respond to UC disorder in the shared household environment [18, 21]. We design a second matched cohort of healthy couples to adjust for the confounding effect on UC‐induced microbial plasticity due to random fluctuations.

As a complex trait, microbial response to UC disorder may involve many factors. We argue that such phenotypic plasticity of one microbe is mediated by its co‐existing microbes in the gut through complex interaction networks. Although correlation‐based networks and Bayesian networks have been widely used as a tool to characterize the pattern and distribution of microbial interactions within the gut microbiota [22, 23], they are limited in terms of simultaneously revealing interaction direction, sign, and strength. We develop a generalized statistical mechanics model for inferring informative, dynamic, omnidirectional, and personalized networks (idopNetworks) from static abundance data [24, 25, 26, 27, 28, 29]. This model integrates evolutionary game theory [30], predator‐prey dynamics [31], and graph theory [32] into a unified framework by which microbial abundance and microbial interactions are mapped as nodes and edges, respectively, in networks. This model is expanded by integrating an allometric scaling law to build a system of quasi‐dynamic mixed ordinary differential equations (qdMODEs), allowing idopNetwork to be reconstructed without the need for longitudinal abundance data. The model further introduces developmental modularity theory and high‐dimensional statistical modeling to coalesce all gut microbes into multi‐layer, multiplex, sparse interactome idopNetworks, under which the fundamental question of how UC‐induced plasticity of each and every microbe is determined by its counterparts can be addressed.

We apply the new model to reconstruct idopNetworks for phenotypic plasticity of individual microbes in response to UC. Metagenomic studies have consistently found compositional shifts in the UC gut microbiota, characterized by an increase in pro‐inflammatory taxa (e.g., *Veillonella*, *Proteobacteria*) and a decrease in beneficial taxa (e.g., *Firmicutes*, *Faecalibacterium prausnitzii*) [33, 34, 35, 36, 37]. Our model can reveal how these compositional changes are formed and influenced by idopNetworks. By comparing how these compositional changes vary between the UC cohort and healthy cohort, our detailed analysis by the new model can better understand the mechanistic basis of dysbiosis in UC.

## 2 Results

### 2.1 Phenotypic Plasticity of the Gut Microbiota

We identify 5 048 bacterial species within the gut microbiota from 24 paired couples, of which half are from UC‐diseased families and the other half from healthy families. We view each host's gut as an ecosystem composed of these diverse microbial species. We calculate the sum of the abundance of all microbes for each host and define it as the habitat index (HI), reflecting the overall “productivity” of the ecosystem. Two spouses from the same family share the common living environment, whose difference in microbial abundance reflect UC‐induced phenotypic plasticity(uPP) if one spouse is an individual with UC and the other is healthy, or fluctuation‐induced phenotypic plasticity (fPP) if both spouses are healthy. We find that, compared to fPP, uPP is greater (p < 0.05) and also much more variable among couples for the HI, suggesting that the gut microbiota as a whole is sensitive to UC disorder, with the extent depending on family environment (Figure 1). Specifically, many microbes acting as opportunistic pathogens are more plastic in response to UC than to fluctuations (uPP > fPP), whereas commensal microbes, such as *Butyrdiaplsyivibrio* and *Eubacterium spp*., display an inverse pattern, i.e., uPP < fPP (Figure 2A).

**FIGURE 1:**
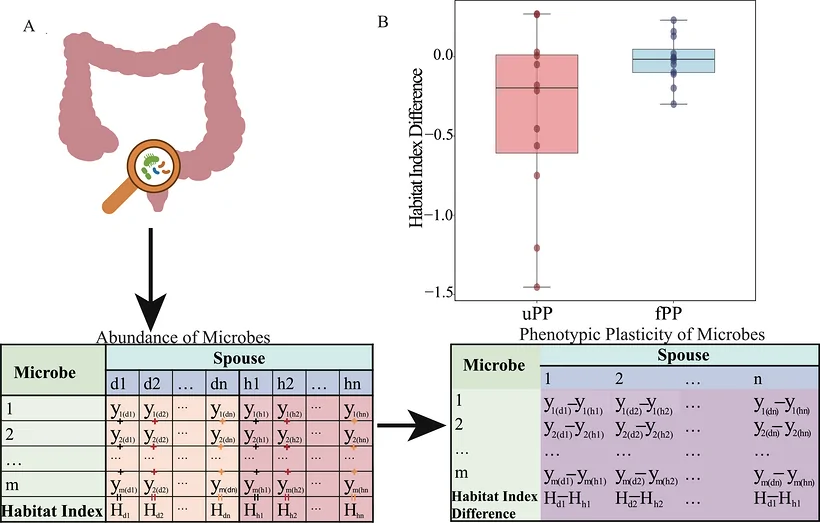
The gut microbiota as a complex system. (A) Data structure of microbial abundance in ulcerative colitis (UC)‐diseased and healthy guts from a spousal pair cohort, from which phenotypic plasticity of microbial abundance is calculated (created with BioRender.com). (B) Boxplots of habitat index for UC patients and healthy subjects.

**FIGURE 2:**
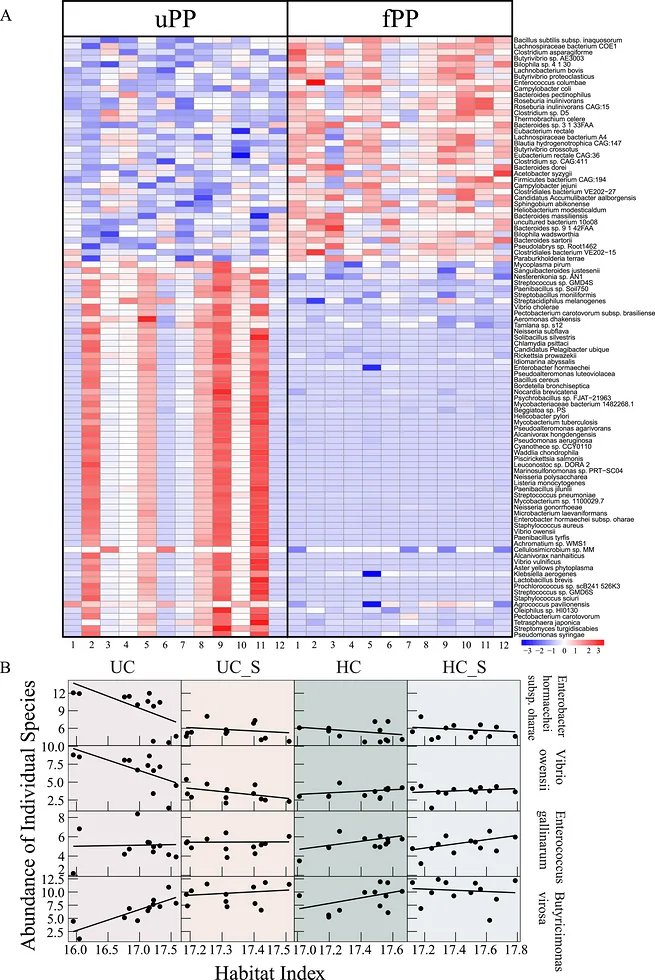
Identification of microbial phenotypic plasticity. (A) Heatmap showing UC‐induced phenotypic plasticity (uPP) and fluctuation‐induced phenotypic plasticity (fPP) of habitat index and species‐level microbial abundance. (B) The fitness of a power equation to the abundance of four randomly selected microbes from UC patients and their healthy spouse (UC_S) from one cohort and two healthy spouses (HC and HC_S) from the second cohort across habitat index. Each dot represents the normalized abundance of a microbe measured, and the black line represents the fitted curve.

The abundance of each microbial species against the HI is a part‐whole relationship, which can be well described by the power‐law equation (Figure 2B). Based on the shape of power curves, we identify four representative patterns of phenotypic plasticity of microbes in response to environmental variation (Figure 2B). The first pattern, termed *disease‐driven plasticity*, is exemplified by *Enterobacter hormaechei subsp. Oharae*, which displays a UC‐specific HI‐varying trajectory that is distinct from the other three groups. The second pattern is (*disease* + *environment*)*‐driven plasticity*, as observed for *Vibrio owensii*, which displays differences in abundance trajectory between UC and healthy spouses from the diseased families (due to the disease) as well as between the UC spouse from the diseased families and two spouses from the healthy families (due to the environment). The third pattern, designated *fluctuation‐driven plasticity*, is represented by *Enterococcus gallinarum*, which exhibits similar trajectories between two spouses from UC families but different trajectories between two spouses from healthy families. The fourth pattern, referred to as (*disease* + *fluctuation*)*‐driven plasticity*, is illustrated by *Butyricimonas virosa*, which exhibits distinct trends between two spouses from both diseased and healthy families. Next, we attempt to identify key microbes that mediate each of these phenotypic plasticity patterns through reconstructing idopNetworks.

### 2.2 Detection of Microbial Modules by Wu's Functional Clustering

The gut microbiota comprises thousands of microbial species that co‐occur within large‐scale ecological networks. However, it is computationally impossible to reconstruct networks that cover all pairwise interactions among 5 048 bacterial species in the gut microbiota. Also, in life systems, such fully interconnected networks never occur [38, 39] per modularity theory, which states that robust and stable large‐scale networks are organized into distinct communities in which members are more functionally related to each other than to those outside the module [40, 41, 42, 43]. As such, we implement Wu's bivariate functional clustering [44, 45] to classify all microbial species into distinct modules based on the similarity of their phenotypic plasticity in response to UC and fluctuations.

We find that 5 048 species are classified into eight modules (coded as M1–M8) according to the BIC criterion (Figure 3A). Each module displays a cohort‐variant pattern of phenotypic plasticity over the HI, implying the impact of the host's inflammatory status on the phenotypic plasticity of microbes. Among the eight modules, M1 shows the most pronounced difference in the HI‐varying trend of phenotypic plasticity between diseased and healthy cohorts. For the healthy cohort, fPP of M1 remains small and stable, with a nearly zero slope in the fitted curve, suggesting highly consistent behavior between healthy individuals. In contrast, in the UC‐diseased cohort, the fitted curve of uPP displays a markedly steeper slope, with values being much greater than those in the healthy cohort. This contrast suggests that module M1 is robust under healthy conditions but becomes markedly responsive to the UC disease environment (Figure 3A).

**FIGURE 3:**
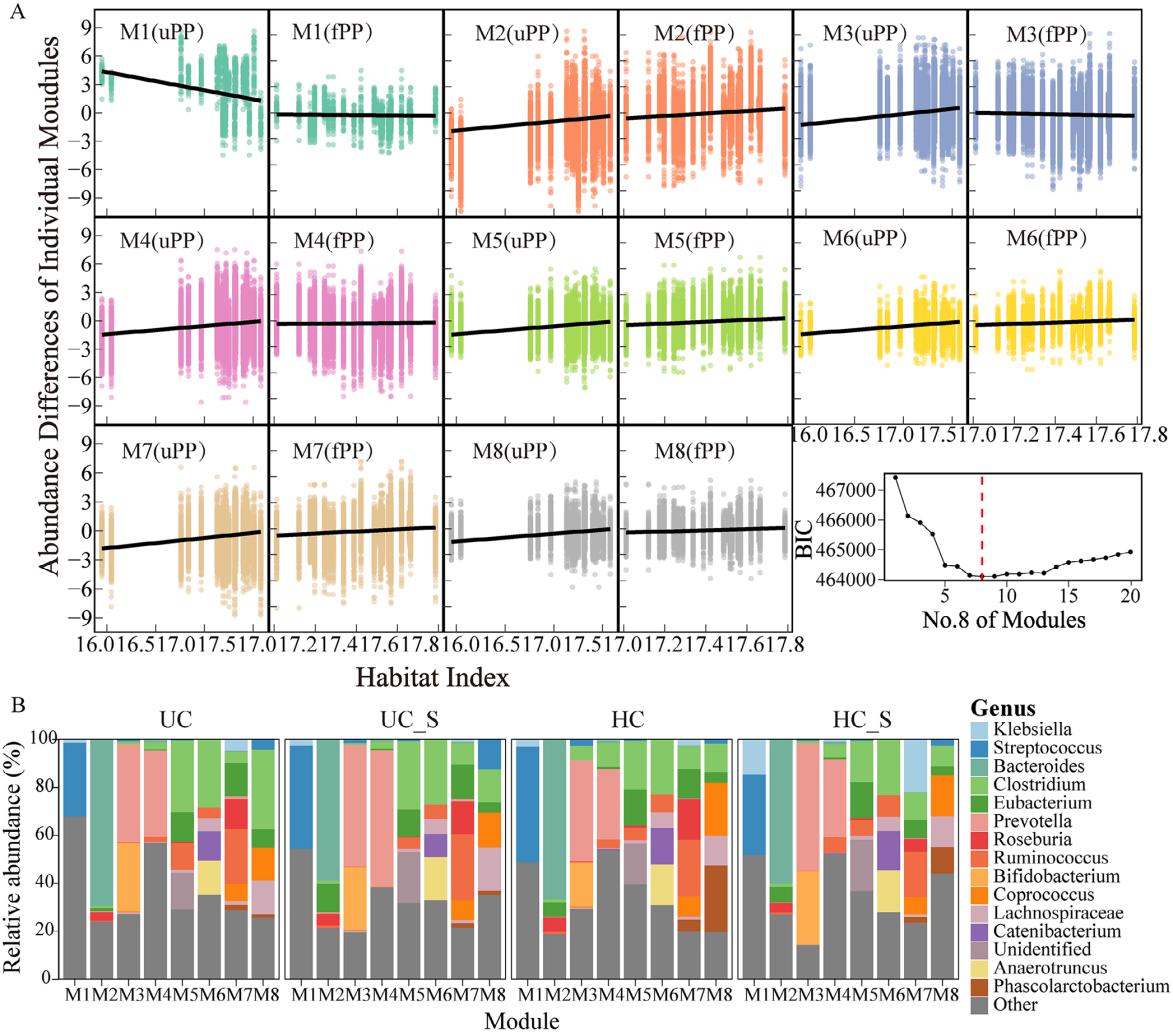
Identification of network communities from a large‐scale network. (A) A total of 5048 microbes classified into eight distinct modules based on similarity.UC‐induced phenotypic plasticity (uPP) and fluctuation‐induced phenotypic plasticity (fPP) across habitat index by bivariate functional clustering. The black line represents the mean trajectory of each module, while the semi‐transparent points denote uPP and fPP values. (B) Microbial components of eight modules at the genus level in four groups (UC vs. UC_S and HC vs. HC_S).

The microbial components identified at the genus level are illustrated in (Figure 3B). M1 is predominantly *Streptococcus*, M2 is dominated by *Bacteroides*, M3 and M4 are mainly composed of *Prevotella*, M5 is primarily represented by *Clostridium* and *Eubacterium*, M6 largely consists of *Clostridium* and *Anaerotruncus*, M7 is dominated by *Ruminococcus*, *Roseburia*, and *Eubacterium*, and M8 is mainly composed of *Clostridium*, *Lachnospiraceae*, and *Coprococcus*. The annotated results of microbiota at taxonomic guilds are consistent with biological interpretation, demonstrating the reliability of functional clustering. The relative abundance of microbes within each module varies among the two spouses from both diseased and healthy cohorts, with the relative abundance of *Eubacterium* (mainly *Eubacterium rectale* and related strains) in module M2 being significantly lower in the UC group compared to the other three healthy groups. In contrast, the abundance of *Ruminococcus* (primarily *Ruminococcus gnavus*) in module M5 is the highest in the UC group. The elevated abundance of *R. gnavus* in IBD patients has been consistently reported in previous studies [46, 47]. Together, these results suggest that the UC disease environment, posing a strong ecological selective pressure, drives a reorganization of the relative abundances of key microbes within the infected guts. Different species appear to adopt distinct response strategies to this environmental perturbation: pro‐inflammatory taxa, such as *R. gnavus* in M5, may better adapt to or even thrive in the inflammatory environment, whereas protective probiotics, such as *E. rectale*, in M2 are suppressed.

To provide additional functional annotation for these modules, we summarized the reported functions of the dominant genera in Table S3. Briefly, the Streptococcus‐dominant M1 may reflect oral‐associated or inflammation‐related features, as oral–gut axis studies have discussed the potential involvement of oral‐associated bacteria in IBD‐related intestinal inflammation [48, 49]. The Bacteroides‐dominant M2 may be associated with carbohydrate utilization, as human gut Bacteroides and Parabacteroides species have broad polysaccharide‐utilization capacity [50]. The *Prevotella*‐dominant M3 and M4 may be related to carbohydrate metabolism, but *Prevotella* has species‐dependent effects and should not be interpreted as uniformly beneficial or harmful [51, 52]. Modules enriched in *Roseburia*, *Eubacterium*, *Lachnospiraceae*, and *Coprococcus* may suggest anaerobic fermentation and SCFA‐related functional features [53, 54], while modules containing Ruminococcus should be interpreted cautiously because R. gnavus has been linked to IBD‐related conditions and inflammatory polysaccharide production [55, 56].

### 2.3 Multilayer Interactome Networks From Healthy to UC‐Diseased States

Wu's functional clustering [44, 45] can not only identify distinct microbial modules for the gut microbiota, but can also be integrated with qdMODEs to frame a bottom‐up approach for subnetwork detection from a complete virtual idopNetwork [26]. While each module represents a subnetwork, we implement Wu's qdMODEs to reconstruct inter‐module coarse‐grained networks using the total abundance level of all microbes within modules. We use a similar approach to reconstruct within‐module fine‐grained networks from the abundance levels of individual microbes. Coarse‐ and fine‐grained networks are linked to form four multilayer, multiplex, multiscale, and multifunctional idopNetworks (4MidopNetworks) for 5 048 microbial species from the gut microbiota for each spouse type in two cohorts (Figure 4). Such 4MidopNetworks provide a complete landscape of how each microbe interacts with every other microbe and how each type of interaction is associated with UC‐ and fluctuation‐induced phenotypic plasticity.

**FIGURE 4:**
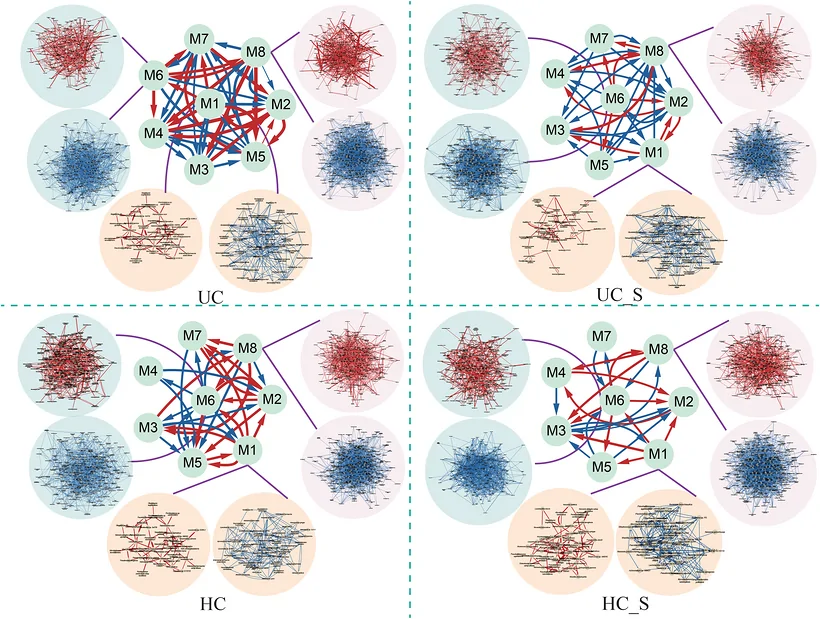
Context‐specific microbial networks reconstructed from 5,048 microbes.

All microbes are integrated into a two‐layer idopNetwork: the central panel depicts the coarse‐grained module–module network (M1–M8), while the peripheral panels show the fine‐grained microbial networks with the names of individual microbes. For each group (UC, UC_S, HC, and HC_S), only the hub module is displayed; the microbial‐level network for each hub is presented as two separate subnetworks (promotion and inhibition). Red and blue lines represent promotion and inhibition, respectively, with the thickness of lines proportional to the strength of interaction.

We find that 4MidopNetworks of four spouse types have varying topological architectures, showing the complexity of microbial interactions in the gut. It is interesting to note that hub modules in the coarse‐grained networks vary between the UC patients and the other three healthy spouses, regardless of the influence of fluctuation. While modules M1 and M8 serve as hubs in the coarse‐grained networks of the UC patients, a hub in the healthy coarse‐grained networks is consistently module M6. This difference suggests that the core structure of the microbial community is reconfigured in the disease state of UC (Figure 5). Inter‐modular interactions also exhibit significant plasticity from healthy to diseased states. In the three healthy spouses, M1 establishes a prey‐predator relationship with M3 (with the former promoting the latter but the latter, M3, suppressing M1), whereas the interaction between M1 and M3 is shifted to an amensalistic relationship in the UC group. Also, M1 promotes M2 in the three healthy groups, but M1 becomes inhibitory to M2 in the UC group.

**FIGURE 5:**
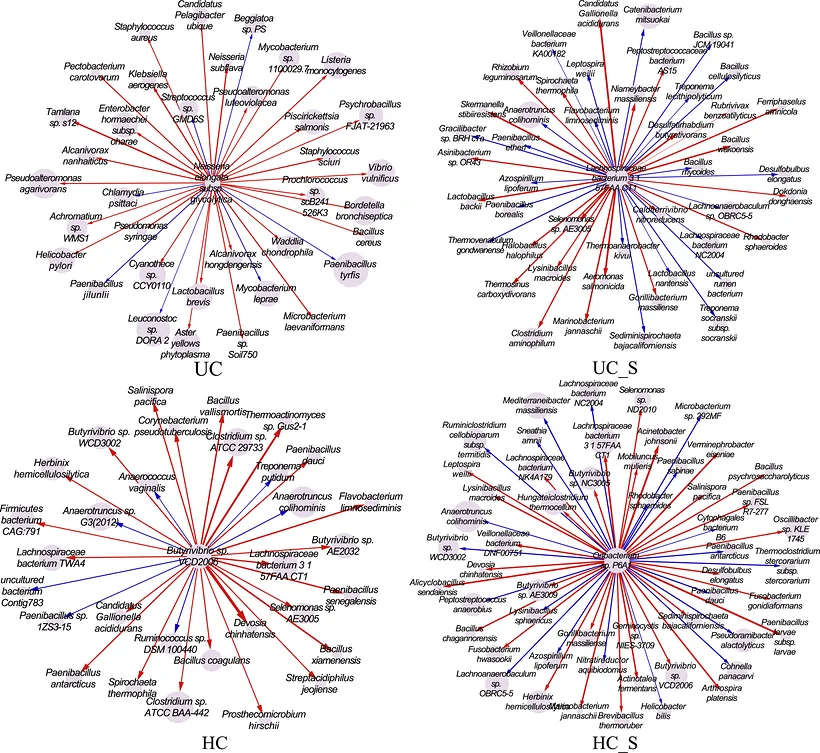
Interaction network of the hub microbe and its associated microbes. Red and blue lines represent promotion and inhibition, respectively, with the thickness of lines proportional to the strength of interaction.

We reconstruct fine‐grained idopNetworks among different microbial species from each module, finding tremendous discrepancies between the diseased and healthy groups. Notably, the functional role of hub species in the fine‐grained idopNetworks diverges markedly between the UC and healthy states (Figure 5). In the non‐UC spouse's fine‐grained network of hub module M6, *Lachnospiraceae bacterium 3_1_57FAA_CT1* functions as a hub that promotes probiotics like *Lactobacillus backii*, while inhibiting detrimental species, such as *Desulfobulbus elongatus*. Similarly, distinct hub species detected in the healthy spouses’ fine‐grained networks from hub module M6, i.e., *Butyrivibrio sp. VCD2006* and *Oribacterium sp. P6A1*), facilitates the growth of various probiotic microbes.

In contrast, the hub species in the UC fine‐grained networks of hub module M1, *Neisseria elongata subsp. glycolytica*, promotes the growth of multiple opportunistic pathogens, including *Klebsiella aerogenes* and *Listeria monocytogenes* (Figure 5). Another hub module, M8, in the UC spouse, *Streptomyces varsoviensis*, suppresses probiotics, such as *Lactobacillus aviarius* and *Lachnospiraceae bacterium AD3010*, while also inhibiting microbes, like *Enterococcus avium* and *Citrobacter freundii*. These results indicate that although specific hub species executing protective functions differ between healthy spouse types, they consistently tend to promote beneficial bacteria. Conversely, under UC‐diseased conditions, hub species are inclined to support opportunistic pathogens.

### 2.4 Disentangling How Microbial Interactions Mediate Phenotypic Plasticity

The merit of qdMODEs is that they can decompose the observed abundance of microbes into their independent components resulting from their intrinsic capacity and dependent components due to influences of other microbes on them (Figures 6 and 7). Module M1 does not distinguish between healthy groups but is much richer in the UC group than in healthy groups, suggesting that it is a predictor of UC risk. Indeed, its independent component in UC patients is considerably larger than what is observed because it is inhibited by module M5, despite slight promotion by M2 (Figure 6). This indicates a stronger intrinsic capacity of M1 to express its ecological function in the UC environment, but it can be canceled by the negative regulator M5. M6 is a hub module of UC spouse's coarse‐grained network, whose independent component is canceled out by a big regulator M7, leading to a reduced observed level of microbial abundance in UC patients. This module displays a relatively high observed level of microbes in three healthy spouse groups because it is influenced, to a much lesser extent, by other modules.

**FIGURE 6:**
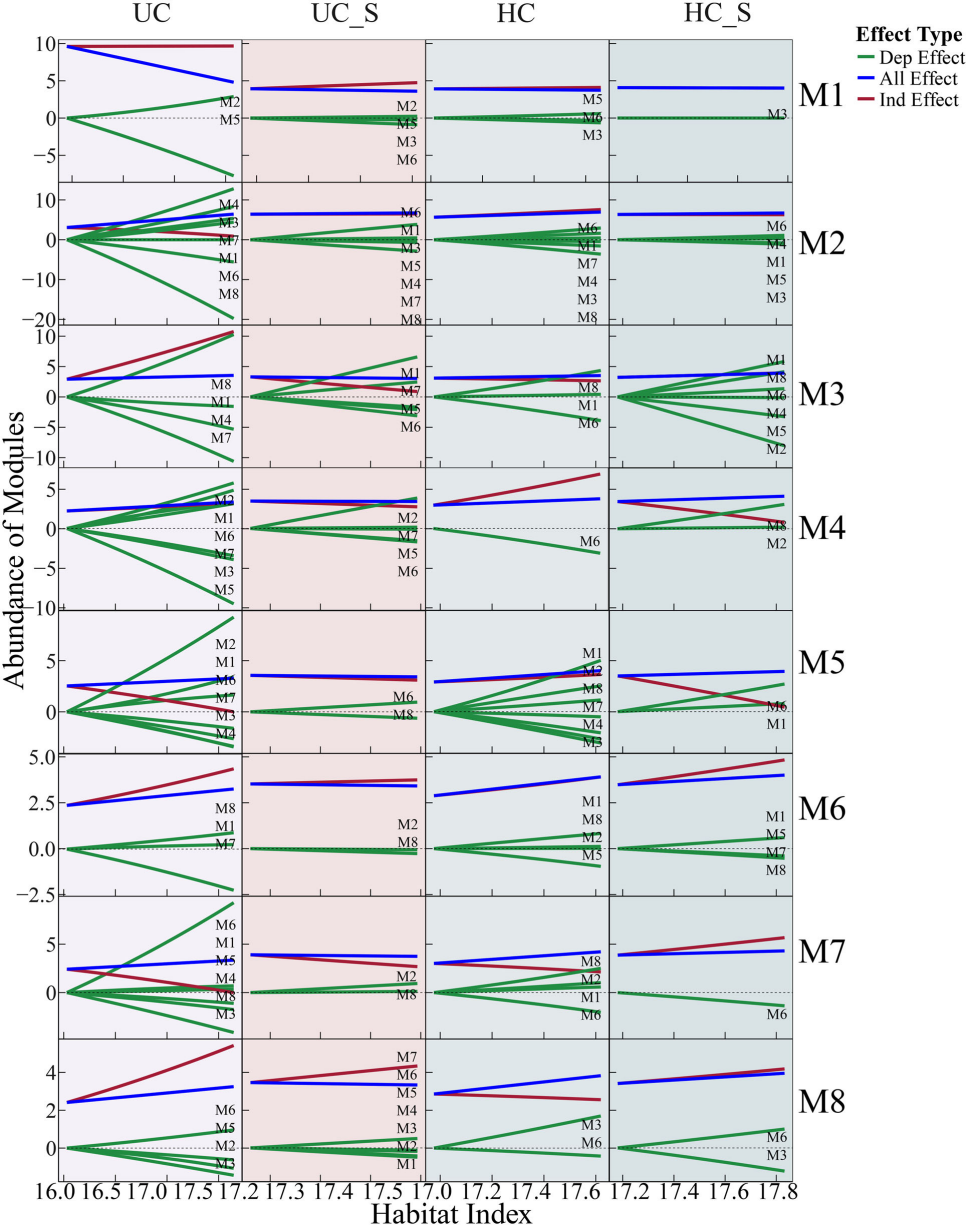
Net total microbial abundance curves of each module (blue line) decomposed into independent component curves resulting from intrinsic capacity (red line) and dependent component curves due to regulation by other modules (green line).

**FIGURE 7:**
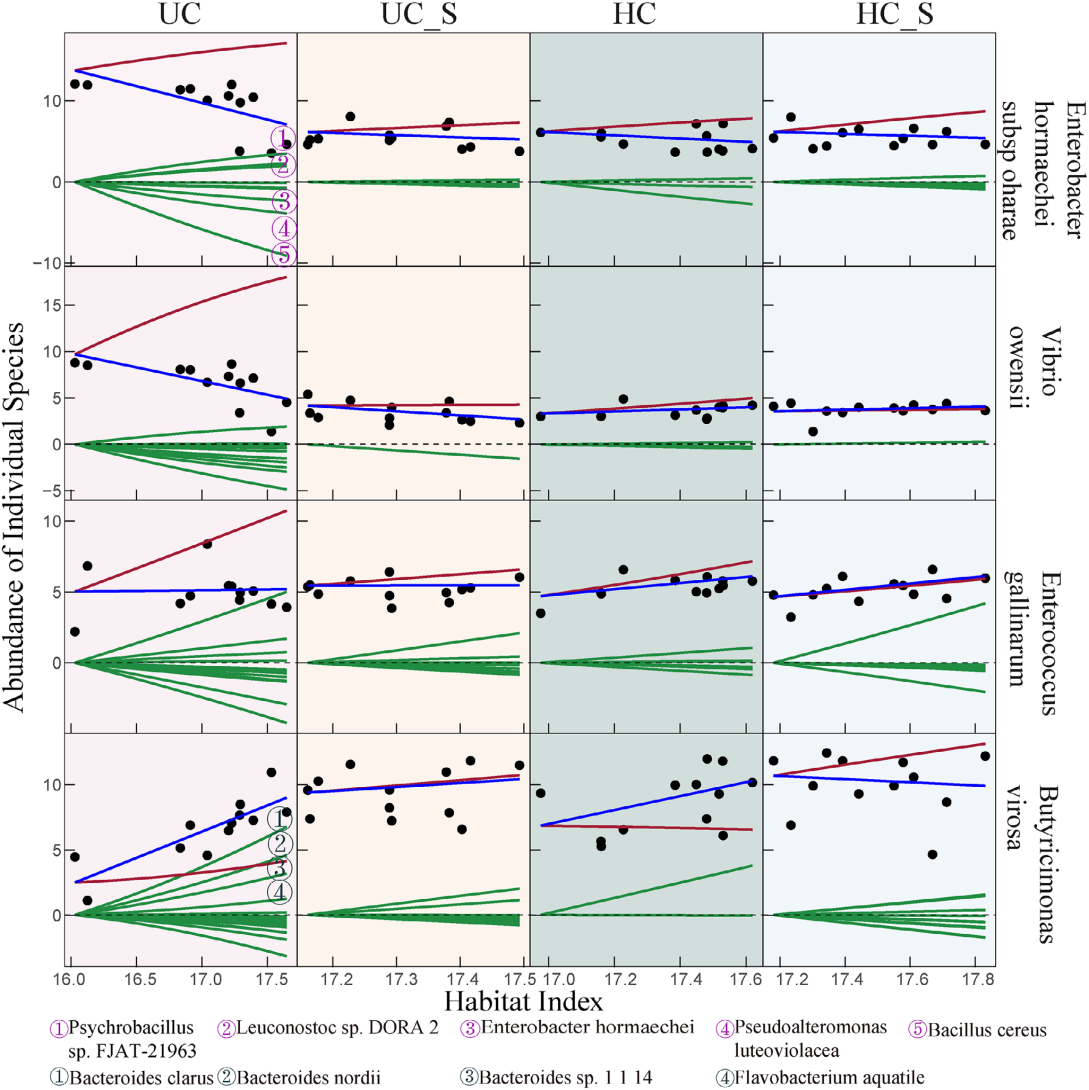
Net total microbial abundance curves of each microbe described in Figure 2B (blue line) decomposed into independent component curves resulting from intrinsic capacity (red line) and dependent component curves due to regulation by other modules (green line).

The key pathogens in the gut microbiota display much more complex interaction architecture (in terms of the number of regulators, interaction sign, and interaction size) with other species for the UC group than for the three healthy groups (Figure 7). *Enterobacter hormaechei subsp. Oharae* is an opportunistic and nosocomial pathobiont in IBD by potentially disrupting gut barriers and increasing inflammation [57]. The uPP of this species, i.e., an increased abundance level from a healthy state to a UC state, is attributed to its strong intrinsic capacity (leading to a high independent component value) induced under the diseased condition, subject to a mix of regulation: positive regulation by *Psychrobacillus sp. FJAT‐21963*, and *Leuconostoc sp. DORA 2* and negative regulation by *Enterobacter hormaechei*, *Pseudoalteromonas luteoviolacea*, and *Bacillus cereus*. As the biggest negative regulator, *B. cereus* plays a dual role in causing gastroenteritis and alleviating IBD symptoms by reducing inflammation and repairing the gut barrier [58]. The decomposition analysis of idopNetworks shows that this species can inhibit *E. hormaechei subsp. Oharae* and, therefore, modulate the gut microbiota and metabolism. A similar trend is observed for *Vibrio owensii* and *Enterococcus gallinarum*, both of which also display a high level of independent component under the UC condition.

*Butyricimonas virosa* is a pathogen associated with IBD [59], displays a lower level of microbial abundance under the UC condition than under the healthy condition (Figure 7). This may be due to its lower intrinsic capacity under the UC condition. However, this pathogen is promoted by several species, making its observed abundance level higher than that of its independent component. Taken together, based on how the phenotypic plasticity of pathogenic species in response to the alteration of health state is affected by their coexisting microbes, we can design an optimal intervention strategy to control these pathogens.

### 2.5 GLMY Dissection of Plasticity‐Related Networks

To analyze complex microbial IDOP networks, we implement GLMY homology theory, pioneered by S.‐T. Yau and his team [60, 61, 62], which is a powerful branch of mathematical topology, moving beyond traditional graphs by studying directed paths and higher‐order structures to reveal intrinsic network properties. It extends classical homology to directed graphs, capturing quantitative and structural information through cycles and higher‐dimensional features, with persistent GLMY homology tracking these features across network filtrations [29, 63, 64, 65]. GLMY theory is used to map information flow initiated from individual microbes within microbial networks at both the module and microbe levels. We estimate the ranks of GLMY homology at dimensions zero, one, and two, and do not identify significant differences in the homology of M6 and M8 networks across dimensions among the four spouse types (Figure 8A). In contrast, the M1 network exhibits a pronounced discrepancy in homology at dimension two between UC and healthy groups.

**FIGURE 8:**
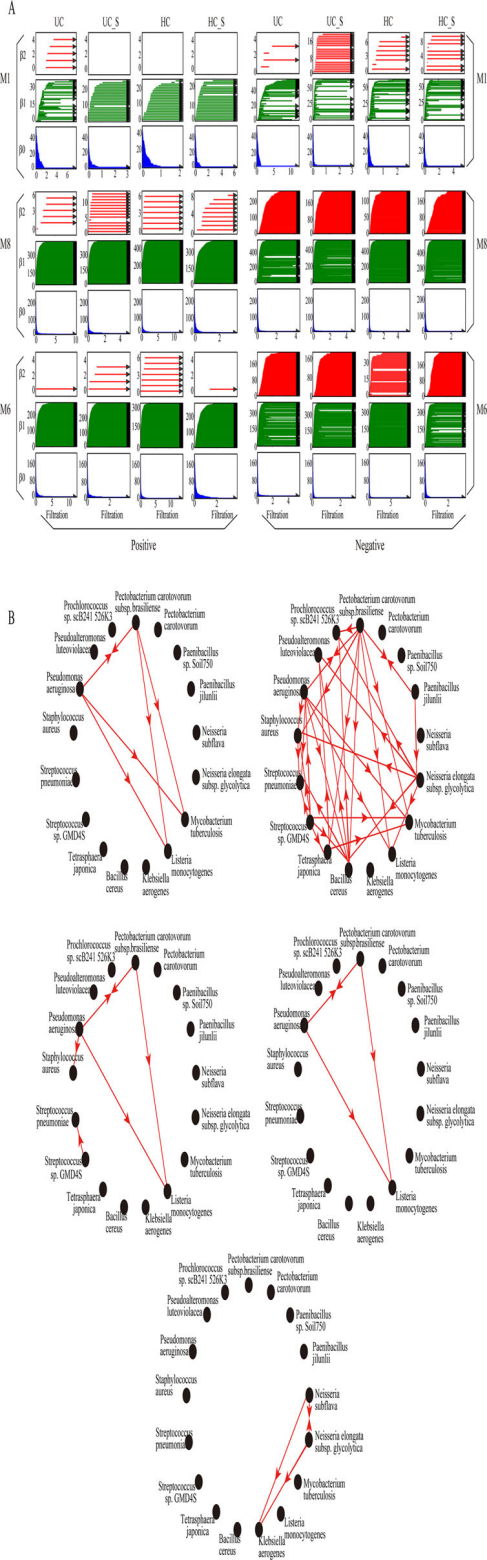
GLMY homology analysis of fine‐grained networks for modules M1 served as a hub for UC groups and M6 as a hub for the HC group. (A) y‐axis represents the ranks of the homology groups (Betti numbers) at dimensions zero, one, and two, and the x‐axis is the filtration, i.e., the change of thresholds for the strength of interaction, separately for positive interaction (promotion) and negative interaction (inhibition). (B) Detailed topological dissection at the β2 level of microbial networks for the four groups. As an example, the dissection is focused on M1's fine‐grained network based on positive interactions.

In positive edge‐derived subnetworks of M1, the UC group contains five topological features at dimension two, while such features were absent in the other three groups. The UC group also shows more one‐dimensional loops than the other groups. These two‐dimensional topological features emerge when the health state changes from healthy to diseased. Among the five two‐dimensional features formed in the UC network, four shared a circular connectivity pattern, where a bidirectional edge between *Pectobacterium carotovorum subsp. Brasiliense* and *Pseudomonas aeruginosa* linked to *Listeria monocytogenes* (Figure 8B). These findings indicate that under the UC condition, these conditional pathogens do not act independently but form tightly interconnected cooperative alliances, resulting in a functionally enhanced mutual promotion network.

In negative edge‐derived subnetworks, more pronounced differences in homology are observed at dimensions one and two among the four spouse groups. The UC network contains 54 holes at dimension one, while the healthy groups have 60, 63, and 62, respectively. At dimension two, the UC network has four voids, compared to 21, 8, and 10 in the three healthy groups. Since increased Betti numbers are generally associated with greater network complexity, these results suggest that disease‐positive networks are more complex than healthy positive networks, while healthy negative networks exhibit higher complexity than disease negative networks.

### 2.6 Biomarker Potential of Phenotypic Plasticity in UC Prediction

To evaluate the predictive performance of each module for UC disease, we build a machine learning model. The results demonstrate that module M1 exhibits the highest predictive power, with an area under the receiver operating characteristic curve (AUC) of 0.82 (Figure 9A). This finding indicates that the spousal difference in microbial abundance based on module M1 effectively distinguishes UC patients from non‐UC individuals, including their healthy spouses and matched healthy families. These results suggest that the differential abundance pattern observed in module M1 between UC patients and their spouses is specific to the disease (Figure 9A).

**FIGURE 9:**
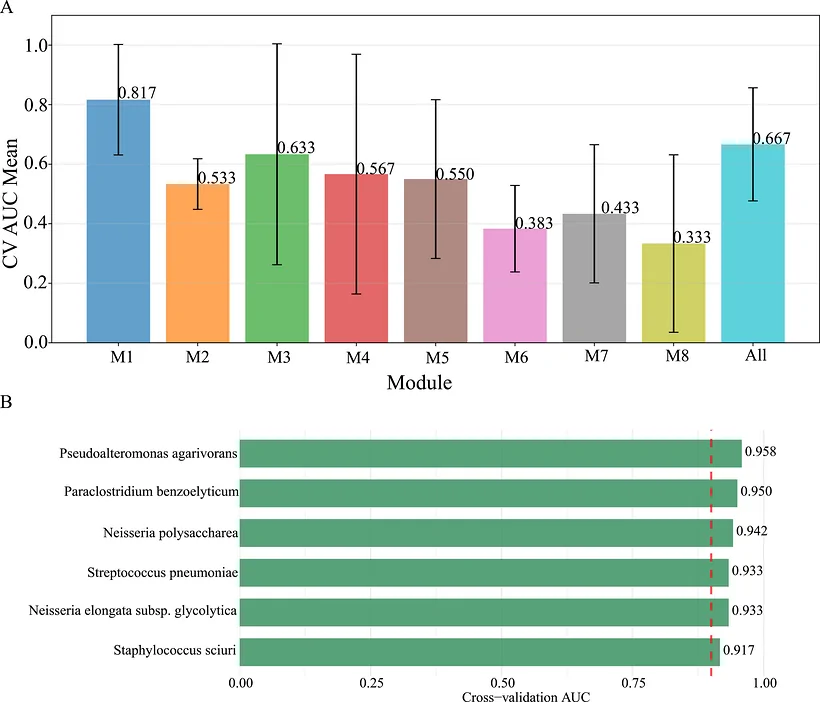
Predictive performance based on microbial uPP. (A) Module‐wise predictive analysis demonstrates that M1 provides the strongest predictive signal. (B) Microbe‐level prediction within M1, in which taxa with cross‐validated AUC > 0.9 were identified and assessed individually, highlighting their substantial contributions to prediction performance.

To evaluate the diagnostic potential of individual species within module M1 for UC, we constructed machine learning classifiers based on the abundance data of each species. The results revealed that six species exhibit an outstanding discriminatory power, each achieving an AUC of greater than 0.9. This indicates that these species can each serve as a robust single biomarker for predicting UC (Figure 9B). These findings suggest that the microbial plasticity signatures identified by the network model contained disease‐related discriminatory information within the present cohort.

## 3 Discussion

This study advances the understanding of ulcerative colitis (UC)‐associated dysbiosis by characterizing microbial phenotypic plasticity at a systems level. Leveraging a powerful idopNetwork model to a spouse‐pair cohort design, we reveal that the UC state is associated with a fundamental restructuring of the gut microbial interactome, which appears to drive a concerted shift in its adaptive ecological dynamics. These findings highlight the adaptive responses of the microbiome to inflammation and provide a new systems‐level perspective on UC‐associated dysbiosis.

Moving beyond conventional microbiome studies that primarily catalog static compositional differences in UC individuals [66, 67], we focus on the overlooked dimension of phenotypic plasticity—the microbial community's dynamic adaptive capacity [68, 69, 70, 71, 72, 73]. Direct measurement of plasticity through longitudinal sampling is hindered by practical constraints and inter‐individual heterogeneity [74, 75, 76]. To overcome this, we design a spouse‐pair cohort in which each couple, one being UC‐diseased and the other being healthy, shares the same household environment so that the difference in microbial abundance between the spouses (uPP) represents the phenotypic plasticity of each microbe in response to UC dysbiosis. We design a second spouse‐pair cohort as a reference involving healthy couples, whose difference in microbial abundance (fPP) is due to fluctuations. Thus, by using fPP to control for shared environmental confounders, we can accurately characterize uPP that results from plastic response to UC dysbiosis.

Based on the spouse‐pair design, we reconstruct idopNetworks to reveal how uPP is mediated by microbial interactions and how host inflammation restructures the adaptive landscape of the gut microbiota, representing a shift in research paradigm from cataloging composition to deciphering the dynamics of the ecological interactome. We show that the UC state is associated not only with an altered taxonomic composition but also with a fundamental restructuring of the gut microbiota's phenotypic plasticity—the operating rules governing microbial responses to environmental variation.

We implement Wu's functional clustering to identify eight modules of phenotypic plasticity, representing coordinated microbial response units under host environmental influence. Notably, M1—dominated by conditionally pathogenic genera, such as *Listeria* and *Pseudomonas*—exhibits markedly enhanced plasticity in response to UC. Previous studies have consistently documented expansion of pathobionts and depletion of beneficial taxa in UC [33, 77, 78]. Thus, increased plasticity of conditional pathogens may serve as a critical driver of the characteristic UC‐associated dysbiosis. Our modeling results show that UC does not merely alter plasticity at the single‐taxon level, but also it restructures plasticity at the module level, indicating a system‐wide ecological reorganization unique to the disease environment. By constructing multilayer interaction networks, we further uncover the broader ecological implications of plasticity [79]. At the coarse‐grained level, the core network module shifted from M6 in healthy subjects to M1 in UC patients, representing a striking hub module shift. This indicates that a network originally centered on beneficial genera—including *Clostridium*, *Anaerotruncus*, *Ruminococcus*, and *Lachnospiraceae*—is replaced in UC by a hub dominated by conditionally pathogenic taxa.

The coarse‐grained network analysis further reveals directional reversals in ecological interactions. For example, the influence of M1 on M2 shifted from positive in the healthy state to negative in the UC state, and the effect of M6 on M2 also flips from facilitative to suppressive. As M2 is enriched for UC‐associated Bacteroides, this highlights that inter‐module ecological interactions (competition vs facilitation) are highly plastic and dynamically reconfigured by the host disease state. Such large‐scale network restructuring likely reflects an adaptive response to pathological pressure, but may simultaneously destabilize the system by altering metabolic outputs and functional connectivity, ultimately exacerbating disease progression. At the fine‐grained level, we also detect substantial rearrangement of interaction structures, consistent with prior work on plasticity‐driven interaction dynamics [80].

Ecological network plasticity can profoundly influence global structural and functional properties [81]. Through GLMY topological analysis [60, 61, 62], we identify UC‐specific high‐dimensional network features. In UC, positive regulatory interactions are markedly more complex, with multiple β_2_ cavity structures emerging within the M1 module, which are entirely absent in healthy individuals. These high‐dimensional features are formed by mutually reinforcing loops among *Listeria monocytogenes*, *Pseudomonas aeruginosa*, and other pathobionts, suggesting that these taxa may cooperatively enhance their adaptation and colonization under UC conditions. Together with the literature linking UC to *Listeria monocytogenes* [82], these findings provide strong ecological and pathophysiological relevance. Overall, our multi‐layer analysis reveals that UC drives a profound reorganization of microbial plasticity, offering a mechanistic framework that moves beyond simple abundance changes. We propose that pathobiont‐centered plasticity enhancement may be a key force underpinning UC‐associated dysbiosis.

Machine learning models incorporating plasticity features demonstrate that high M1 plasticity has a strong predictive value for UC (AUC = 0.82), suggesting the feasibility of developing plasticity‐based biomarkers. Several taxa within M1—such as *Enterobacter hormaechei* (high virulence factors), *Klebsiella spp*. (colitogenic potential), and *Klebsiella aerogenes* (activates intestinal inflammasomes)—also exhibited strong predictive performance [83, 84, 85, 86, 87, 88], consistent with previous IBD literature. These results indicate that plasticity signatures may hold translational potential for diagnosis or risk stratification, although validation in larger cohorts is needed.

By leveraging the idopNetwork model, we have systematically profiled the phenotypic plasticity of the gut microbiota from multiple dimensions and hierarchical layers, offering a mechanistic view of UC‐related dysbiosis that surpasses traditional abundance‐based analyses. However, these interpretations should be made with caution since a relatively small sample size was used. For any robust statistical estimation, adequate sample size is essential. Yet, our idopNetwork model is grounded in fundamental physical principles, such as the allometric scaling law, which requires the mathematical fitting of only 3 to 5 parameters. Previous simulation studies have confirmed that a sample size of *n* = 10–12 could yield robust power for interaction detection while maintaining a low false‐positive rate of less than 10% [89]. As such, despite the modest sample size used, we are still confident in the validity and reliability of our findings.

A key challenge will be to determine how and to what extent microbial plasticity impacts host physiology. This requires integrative investigation of host genetics, immune states, microbial plasticity, and microbial interaction networks, ultimately constructing a host–microbiota–plasticity multilayer framework to capture the ecological pathology of UC more comprehensively. Moreover, the molecular basis of microbial plasticity remains largely unknown. Epigenetic regulation, quorum sensing, and metabolite cross‐feeding are promising mechanistic candidates that deserve further investigation. Extending network analyses to viruses and fungi will also provide a more holistic view of the UC microbiome. Accordingly, future studies integrating functional metagenomics, metatranscriptomics, metabolomics, gnotobiotic models, and in vitro microbial consortia will be needed to validate the functional relevance and clinical utility of these network‐derived findings.

Theoretically, integrating phenotypic plasticity with the idopNetwork framework may enable a cross‐level dynamic prediction system that bridges genes, phenotypes, and community behavior. This strategy could be broadly applicable to other disease models, revealing the general and disease‐specific principles of microbiome plasticity.

## 4 Conclusion

In summary, by developing a novel network framework, we reveal that UC fundamentally enhances the phenotypic plasticity of the gut microbiota, driving a system‐wide ecological reorganization. This rewiring is characterized by the emergence of a conditionally pathogenic hub module (M1), which elucidates a shift in the community's adaptive strategy. The high predictive value of these plasticity signatures not only highlights their biomarker potential but also establishes phenotypic plasticity as a key mechanistic layer in UC pathogenesis, offering a new avenue for predictive and targeted ecological interventions.

## 5 Experimental Methods

### 5.1 The Gut Microbiota as a Complex System

We consider the gut of each individual as an ecosystem where different microbes (say *m*) collectively constitute an ecological habitat. For each habitat, we define the sum of the abundance of all colonizing microbes as the habitat index (HI), which reflects the capacity of the habitat to support microbial survival and proliferation through essential resources. Such habitat capacity may be shaped by a mixture of factors, including host genetics, diet, lifestyle, and nutrient availability [90, 91]. The read‐derived species‐level absolute abundance matrix generated from shotgun metagenomic taxonomic profiling was used for HI calculation. Variation in HI across individual guts may be influenced by microbial biodiversity. Let *y_ji_* denote the abundance of microbe *j* (*j* = 1, …, *m*) from habitat *i*, and HI is then calculated as

(1) $$H_{i}=\sum_{j=1}^{m}y_{ji}\;$$

The abundance of a given microbe and the total abundance of all microbes across samples define a part–whole relationship within an ecological community. Biologically, such a relationship follows allometric scaling theory [92], which states that the size of a part scales disproportionately with respect to the whole. To characterize this relationship, we introduce a power equation describing how the abundance of an individual microbe relates to the total microbial abundance. For habitat *i* and microbe *j*, the relationship between the abundance of microbe *j* and HI can be mathematically expressed as

(2) $$y_{ji}=\alpha_{j}\;H_{i}^{\beta_{j}}\;$$

where *α_j_* and *β_j_* are the proportionality coefficient and allometric exponent of the power equation for microbe *j* expressed in sample *i*. This power equation serializes microbial abundance in discrete guts as a range of continuous habitats, in which sense *y_ji_* can be rewritten as *y_j_* (*H_i_*), expressed as a function of HI. Through this power equation, static abundance data measured once for each individual can be converted into their quasi‐dynamic space.

### 5.2 Quantifying Phenotypic Plasticity in a Spouse‐Pair Cohort

To study the phenotypic plasticity of microbial abundance, we measure each microbe *j* in two different environments. Feng et al. [18]. suggested that differences in microbial abundance between two environments can be used as a proxy for phenotypic plasticity. To investigate how a microbe responds to the change in host human condition from healthy to diseased, we simultaneously measure microbial abundance in a healthy individual and a diseased individual. To control for confounding by environmental factors and lifestyle, we design a spouse‐pair cohort in which multiple pairs of couples (say *n*), each with a diseased patient and a healthy spouse, who share the common household environment, are sampled. Thus, the difference in the abundance of microbe *j* between diseased and healthy spouses from the same family represents disease‐induced phenotypic plasticity, which is expressed, across habitats, as

(3) $$\begin{array}{ccc} z_{j}=\left(z_{j1},\text{…}z_{jn}\right) & = & \left(\left(y_{dj}\left(H_{d1}\right)-y_{hj}\left(H_{h1}\right)\right),\right.\\ & & \left.\text{…}\left(y_{dj}\left(H_{dn}\right)-y_{hj}\left(H_{hn}\right)\right)\right) \end{array}$$

where $\left(z_{j1},\text{…}z_{jn}\right)$ is a vector of disease‐induced phenotypic plasticity for microbe *j* over *n* pairs of couples, and *y_dj_* (*H_di_*) and *y_hj_* (*H_hi_*) are the abundance of microbe *j* in the diseased spouse and healthy spouse, respectively, from the same family *i*. The size and sign of each element in Equation (3) reflect the degree and pattern of how microbe *j* responds to host health change from healthy to diseased.

Because of a shared macroenvironment, **z**_*j*_ can be regarded as being due to the change of host health state. However, it may also include some confounding effects due to microenvironmental fluctuations. To characterize the impact of these confounding effects on **z**_*j*_, we design a second cohort in which multiple matched couples (with both spouses being healthy) are measured for the same set of microbial species in the guts. Similarly, we calculate the difference in abundance of a microbe between the two healthy spouses from the same family and define it as fluctuation‐induced phenotypic plasticity.

### 5.3 The qdMODEs of idopNetwork Inference

In the gut microbiota, different microbes interact with each other to form complex interaction networks. Existing approaches for network inference can only capture the strength of interactions (correlation‐based networks) or the direction of interactions (Bayesian networks) [22, 23], but there is a strong need to reconstruct a network that can characterize all properties of interactions, such as bidirectionality, sign, and weight. Such highly informative networks have been reconstructed by developing a generalized statistical mechanics model [24, 26]. In complex gut microbiota, microbial behavior can be interpreted through the lens of evolutionary game theory [30]. Game theory, originating in economic research [93], postulates that a rational player chooses an optimal strategy to maximize its payoff in response to the strategies of others. This decision process iterates until a Nash equilibrium—representing a strategically stable state—is reached [94]. When combined with evolutionary principles, game theory extends to evolutionary game theory, in which evolutionarily stable strategies correspond to Nash equilibria without requiring microbes to behave rationally [95, 96]. To model microbial interactions, we integrate evolutionary game theory and predator–prey theory [97] into a unified framework to characterize the impact of predation on prey population dynamics. This integration leads to a generalized Lotka–Volterra (LV) framework [98] for describing “strategic” interactions among non‐rational microbes.

This generalized LV framework can be described as a generalized statistical mechanics model that characterizes microbial interactions within the gut microbiota through a system of mixed ordinary differential equations (MODEs). This model can be expanded to map microbial interactions from static abundance data by integrating a power equation to build a system of quasi‐dynamic MODEs (qdMODEs), whose time derivative is replaced by the HI derivative. The qdMODE of microbe *j* decomposes its overall abundance level at a specific individual *i* into the independent component due to the intrinsic strategy of this microbe and the dependent component resulting from the influence of coexisting microbes on it, which is expressed as

(4) $$\frac{dy_{j}\left(H_{i}\right)}{dH_{i}}=f_{j}\;\left(y_{j}\left(H_{i}\right);\Theta_{j}\right)+\sum_{\;j^{\prime }=1,j^{\prime }\neq j}^{m}f_{jj^{\prime }}\left(y_{j^{\prime }}\left(H_{i}\right);\Theta_{jj^{\prime }}\right)\;$$

where the independent component *f_j_* (·) of microbe *j* is fully expressed when it is in isolation and the dependent component *f*_*jj*′_(·) describes the influence of microbe $\;j^{\prime }\left(\;j^{\prime }=1,\text{…},j-1,j+1,\text{…}m\right)$ on microbe *j*. These two types of components may not have explicit forms, so a nonparametric approach, such as Legendre orthogonal polynomials (LOP), is implemented to fit their HV‐varying trajectories [99] using parametersΘ_*j*_andΘ_*jj*′_. These qdMODEs can be solved by implementing a mathematical or statistical algorithm.

Microbial interaction networks can be reconstructed by estimating the independent and dependent components of qdMODEs and encoding the independent estimates as nodes and the dependent estimates as edges into graphs. Full properties of microbial interactions can be captured by *f*_*jj*′_(·), involving which direction one microbe interacts with the other microbe, how strong these interactions are, and whether the interactions present positive or negative regulation. Since qdMODEs are a function of HI, this implies that HIV‐varying dynamic networks can be inferred. Statistical tests can be developed to test whether a particular directed interaction is significant, and this procedure allows each and every active interaction to be detected omnidirectionally hidden in the gut microbiota. Finally, HI can be individualized or merged to be context‐specific, such as healthy networks vs. diseased networks, implying that personalized networks can be reconstructed. Taken together, informative, dynamic, omnidirectional, and personalized networks (idopNetworks) can be reconstructed from any static data of microbial abundance.

### 5.4 Sparse and Modular idopNetworks Fundamental to the Gut Microbiota

In biological systems, it is unlikely that each microbe interacts with every other microbe in the community, as a fully connected network would not support the robustness and stability in response to random perturbations [100, 101, 102]. Consequently, even if a habitat contains a large number of microbes, the interaction networks they form are generally sparse and modular.

### 5.5 Variable Selection for Sparse Networks

For any microbe *j* treated as a response variable, the remaining *m−*1microbes serve as potential predictors, which results in substantial complexity for the entire network. However, network theory suggests that sparse connectivity enhances system stability and resilience to external perturbations [103, 104]. We employ LASSO [105] and its variants [106, 107] to identify the microbes most strongly associated with each microbe. Let *d* (*d* << *m*) denote the number of these most influential microbes for microbe *j*. By replacing *m* with *d* in the second term of Equation (4), the full model is reduced to a sparse model, enabling the reconstruction of a sparsely connected network.

### 5.6 Wu's Functional Clustering for Modular Networks

One strategy to ensure network modularity is to classify all microbes into distinct modules based on developmental modularity theory, which states that entities within a community can be divided into modules whose internal connections are stronger than their connections with other modules [108, 109]. Wu and his team have developed a computational model for functional clustering based on a Gaussian mixture model (GMM). This clustering approach has proven to be powerful for classification using function‐valued data. We implement Wu's functional clustering to divide the whole networks into distinct network communities. Let $z_{j}=(z_{j}\;\left(1\right),\text{…},\;z_{j}\left(n\right)$ denote a vector of microbial plasticity of microbe *j* between two spouses from the same family across *n* habitats. We formulate the GMM‐based likelihood for clustering all *m* microbes into different numbers of modules, which is expressed as

(5) $$L\left(z\right)=\prod_{j=1}^{m}\;\sum_{l=1}^{L}\left[\pi_{l}f_{l}\left(z_{j};\mu_{l},\Sigma \right)\right]\;$$

where *L* is the number of modules, π_*l*_ is a prior probability representing the proportion of module *l*, and *f_l_* (**z**_*j*_; **µ**_*l*_,**Σ**) is an *n*‐dimensional normal distribution with cluster‐specific mean vectors:

(6) $$\begin{array}{ccc} \mu_{l} & = & \mu_{l}\;\left(1\right),\text{…},\mu_{l}\left(n\right))\\ & = & \left(\left(\mu_{l}^{d}\left(H_{d1}\right)-\mu_{l}^{h}\left(H_{h1}\right)\right),\text{…},(\mu_{l}^{d}\left(H_{dn}\right)-\mu_{l}^{h}\left(H_{hn}\right)\right)) \end{array}$$

where $\mu_{l}$ is fitted by power Equation (2), specifically for each couple, expressed as

(7) $$\begin{array}{ccc} \mu_{l}^{d}\;\left(H_{dt}\right) & = & \alpha_{l}^{d}\;H_{dt}^{\beta_{l}^{d}}\\ \mu_{l}^{h}\;\left(H_{ht}\right) & = & \alpha_{l}^{h}\;H_{ht}^{\beta_{l}^{h}} \end{array}$$

and the covariance matrix of phenotypic plasticity of microbes has the following structure:

(8) $$\Sigma =\left(\begin{array}{ccc} \sigma_{1}^{2} & \text{…} & 0\\ \vdots & \ddots & \vdots \\ 0 & \text{…} & \sigma_{n}^{2} \end{array}\right)\;$$

In the calculation, the maximum likelihood estimation (MLEs) of the parameters of the power equation and covariance matrix is performed by a hybrid EM algorithm. In the E step, we calculate the posterior probability that microbe j belongs to a particular module l in each iteration of the algorithm using the following equation:

(9) $$\gamma_{jk}=\frac{\pi_{l}f_{l}\left(z_{j};\mu_{l},\Sigma \right)}{\sum_{l^{\prime }=1}^{L}\pi_{k^{\prime }}f_{l}\left(z_{j};\mu_{l^{\prime }},\Sigma \right)}\;$$

In the M step, we calculate the proportion of module *l* by using

(10) $$\pi_{l}=\frac{\sum_{j=1}^{m}\gamma_{jl}}{m}\;$$

The E and M steps are iterated to obtain maximum likelihood estimates (MLEs) of the parameters in the mean vector and covariance matrix, respectively. The optimal number of clusters is determined using the Bayesian information criterion (BIC). The module with the largest posterior probability for an individual microbe, calculated after the final number of clusters is determined, is the optimal module into which this microbe is classified.

### 5.7 The Reconstruction of Multilayer Networks

Functional clustering classifies all microbes into distinct modules. This clustering partitions the original network into multiple small network communities, within which microbial interactions are tighter than those between communities. The interactions among these communities constitute the coarse‐grained network, whereas the networks reconstructed from the abundance profiles of individual microbes within each community are referred to as fine‐grained networks. The integration of fine‐grained and coarse‐grained networks forms a multilayer network, in which independent effects are represented as nodes and dependent effects as edges. We then analyze the functional characteristics of these networks. In this framework, a link associated with microbe A is considered outgoing if A actively promotes or inhibits other microbes, and incoming if A is passively promoted or inhibited by others. Microbes with a greater number of links exert stronger influence within the network, and those possessing the largest number of outgoing links are defined as hub microbes. Taken together, we multilayer, multiplex, and multiscale sparse idopNetworks, which can systematically characterize the internal workings of the gut microbiota.

### 5.8 GLMY Homology Theory of Digraphs

Given a directed network *𝐺 = (V, E)*, where *V =* {𝑣_1_, ⋯, 𝑣_𝑛_} denotes the node set and *E ⊂ V × V* denotes the edge set. An elementary *p*‐path is any sequence of *p* + 1 nodes of *V*. Taking coefficients in a field F, let Λ_𝑝_ be the vector space spanned by the elementary *p*‐paths of G over F. Define the boundary operator 𝜕_𝑝_: Λ_𝑝_→Λ_𝑝−1_ by the formula below:

(11) $$\partial_{p}\left(v_{i_{0}}\cdots v_{i_{p}}\right)=\sum_{q=0}^{p}{\left(-1\right)}^{q}v_{i_{0}}\cdots v_{i_{q-1}}v_{i_{q+1}}\cdots v_{i_{p}}$$

Moreover, it is necessary to introduce the concept of an allowed path as follows. An allowed *p*‐path is an elementary *p*‐path such that (𝑣_𝑖𝑗_, 𝑣_𝑖𝑗+1_) ∈ 𝐸(0 ≤ 𝑗 < 𝑝). Now let A_𝑝_ be the subspace of Λ_𝑝_ spanned by the allowed *p*‐paths of 𝐺, and consider the following subspace of A_𝑝_:

(12) $$\Omega^{p}=\left\{\sigma \in A_{p}|\partial_{p}\left(\sigma \right)\in A_{p-1}\right\}\;$$

The restriction of the boundary operator 𝜕_𝑝_ of Λ_𝑝_ to Ω_p_ forms a chain complex of homology groups.

(13) $$0\leftarrow \Omega_{0}\overset{\partial_{1}}{\leftarrow }\cdots \overset{\partial_{p-1}}{\leftarrow }\Omega_{p-1}\overset{\partial_{p}}{\leftarrow }\Omega_{p}\overset{\partial_{p+1}}{\leftarrow }\Omega_{p+1}\leftarrow \cdots$$

where 𝜕_𝑝−1_𝜕_𝑝_ = 0. The *p*‐th GLMY homology of 𝐺 is then defined by H_𝑝_(𝐺) = Ker𝜕_𝑝_/lm𝜕_𝑝+1_. The *p*‐th GLMY Betti number of G is the rank of H_𝑝_(G), denoted by 𝛽_𝑝_. It provides a sequence of topological features for directed networks. Here, 𝛽_0_ represents the number of connected components of the underlying undirected network, 𝛽_1_ identifies directed cycle structures up to their interactions, and 𝛽_𝑝_(𝑝 ≥ 2) detects specific higher‐order interacting structures on *p*‐pathways.

By integrating the filtration process of a digraph with GLMY homology, we can construct the persistent GLMY homology. Specifically, start with a filtration of a digraph *G*, which is a sequence of nested digraphs.

(14) $$\emptyset \emptyset =G_{1}\;\subset G_{2}\subset \text{…}\subset G_{n-1}\subset \;G_{n}=G\;$$

where 𝐺_𝑖_ is a subgraph of 𝐺_𝑖+1_ (0 < <*n*). For each digraph 𝐺_𝑖_, considering its *p*‐th GLMY homology 𝐻_𝑝_(𝐺_𝑖_), a sequence of homologies can be derived as

(15) $$H_{p}\left(G_{1}\right)\overset{f}{\to }H_{p}\left(\text{…}\right)\overset{f}{\to }\text{…}\overset{f}{\to }H_{p}\left(G_{n-1}\right)\overset{f}{\to }H_{p}\left(G_{n}\right)\;$$

where *f* is induced from the inclusion between two adjacent digraphs. The evolution of homology along this sequence defines the *p*‐th persistent GLMY homology for the filtration of G. When computed over a field, the resulting persistent GLMY homology can be represented as a persistent barcode or diagram, providing a multiscale topological characterization of the structural properties of the digraph G. Importantly, GLMY persistent homology facilitates the examination of a digraph's topological features across multiple resolution scales.

### 5.9 Discriminative Power Assessment of Microbial Modules

To evaluate the discriminative power of the identified microbial modules, we employ a machine learning framework for UC phenotype prediction. The XGBoost classifier is implemented for this binary classification task to distinguish UC patients (labeled as 1) from healthy controls (labeled as 0). To ensure a robust evaluation, the dataset is split into training and testing sets with an 80:20 ratio, preserving the class distribution through stratification. Model performance is rigorously assessed using fivefold cross‐validation and evaluated by cross‐validated Area Under the Receiver Operating Characteristic curve (CV AUC).

### 5.10 Patient Recruitment and Ethics

We recruited twelve individuals with UC together with their healthy spouses from the Department of Gastroenterology at Beijing Chaoyang Hospital, Capital Medical University. UC diagnosis was established based on standard clinical, imaging, and endoscopic criteria and confirmed by histological examination of colonic biopsies. Twelve additional healthy volunteers were enrolled through the hospital's Health Examination Center and were matched to UC patients by age and sex distribution. Eligible participants had no history of inflammatory bowel disease, irritable bowel syndrome, chronic diarrhea, gastrointestinal malignancy, or other definite gastrointestinal diseases. They also had no history of autoimmune or connective tissue diseases, including rheumatoid arthritis, systemic lupus erythematosus, systemic sclerosis, and Sjögren's syndrome. Participants with uncontrolled cardiovascular, respiratory, hepatic, renal, hematologic, endocrine/metabolic, or neuropsychiatric disorders were excluded, as were those with active infection or recent severe infection. None of the participants had used antibiotics within 3 months before fecal sample collection or received systemic corticosteroids, immunosuppressants, biologics, small‐molecule agents, or other medications that might substantially affect the gut microbiota. All participants were able to complete fecal sample collection and clinical data acquisition and provided written informed consent. Women of childbearing potential had a negative pregnancy test at screening and were not pregnant or lactating, with no plan for pregnancy during the study period. Demographic and clinical characteristics of the study population are summarized in Table S1, and detailed clinical information of the participants is provided in Table S2.

The study was approved by the Medical Ethics Committee of Beijing Chaoyang Hospital, and written informed consent was obtained from all participants.

### 5.11 Sample Collection, Processing, and DNA Extraction

Fresh fecal samples were immediately aliquoted into pre‐sterilized cryovials prefilled with DNA stabilization solution and stored at −80°C until further processing. For DNA extraction, approximately 0.25 g of each fecal aliquot was processed using the PowerSoil DNA Isolation Kit (MoBio Laboratories, 12888) according to the manufacturer's protocol. The resulting DNA was then stored at −80°C in DNase‐free microtubes.

### 5.12 Metagenomics Sequencing

Metagenomic libraries were normalized and sequenced on an Illumina HiSeq 4000 platform (Illumina, San Diego, CA, USA). Raw reads were filtered using Trimmomatic, and trimmed reads shorter than 150 bp were removed. High‐quality reads were assembled using MEGAHIT, and contigs <500 bp were discarded. Open reading frames (ORFs) were predicted using Prodigal in meta mode (‐p meta). Non‐redundant gene catalogs were constructed with CD‐HIT. Clean reads were then mapped to the gene catalog using Bowtie. Predicted genes were annotated against the NCBI NR database using DIAMOND BLASTX.

### 5.13 Statistical Analysis

Statistical analyses were performed using R software version 4.4.3. UC‐induced phenotypic plasticity (uPP) and fluctuation‐induced phenotypic plasticity (fPP) were compared using a two‐sided Wilcoxon rank‐sum test. A two‐sided *p*‐value < 0.05 was considered statistically significant. Module‐level differences were evaluated based on the fitted plasticity trajectories generated by the functional clustering model. These trajectory patterns were considered model‐derived descriptive results, and no separate hypothesis test was performed to compare fitted trajectories among modules. For GLMY homology analysis, Betti numbers across filtration thresholds were used as descriptive topological summaries of the reconstructed directed networks. Variations in homology ranks were therefore interpreted as network‐level topological patterns rather than conventional statistically tested differences.

## Author Contributions

**Huizhen Li**: writing – original draft, formal analysis, visualization, validation, software, data curation. **Jiayi Li**: software, visualization, formal analysis, validation. **Ang Dong**: conceptualization, methodology, software, data curation. **Xiaoyan Guo**: data curation, supervision. **Qiuyue Tu**: supervision, investigation, formal analysis. **Zelin Feng**: formal analysis, supervision, investigation. **Haojia Lin**: supervision, investigation. **Yanru Zhang**: investigation, formal analysis, supervision. **Yuxuan Hao**: supervision, formal analysis, investigation. **Huiying Gong**: visualization, writing – review and editing. **Yihan Meng**: visualization, writing – review and editing. **Yu Wang**: software, methodology. **Shing‐Tung Yau**: methodology, conceptualization, supervision, funding acquisition, project administration. **Xiaocang Cao**: resources, funding acquisition, project administration, writing – review and editing, data curation, supervision. **Rongling Wu**: writing – review and editing, resources, funding acquisition, project administration, supervision, data curation, conceptualization, methodology. **Xinjuan Liu**: writing – review and editing, project administration, resources, funding acquisition, supervision, data curation.

## Conflicts of Interest

The authors declare no conflicts of interest.

## Supporting information




**Supporting File**: advs78043‐sup‐0001‐SuppMat.docx.

## Data Availability

The data and code used in this study have been made publicly available on GitHub (https://github.com/HuizhenLi/GutMicrobiota).
