# Supplementary material for: Omnidirectional Interaction Networks Modulating Phenotypic Plasticity in the Gut Microbiota Towards Ulcerative Colitis
Source: Adv Sci (Weinh). 2026 Oct 6:e78043. Online ahead of print. doi: 10.1002/advs.78043 (PMC13643121; doi:10.1002/advs.78043)
Supplement: Supplementary file 1 [file ADVS-9999-e78043-s001.docx]

Supplementary Table S1. Demographic and clinical characteristics of the study population

| **Variable** | **UC** | **UC_S** | **HC** | **HC_S** |
| --- | --- | --- | --- | --- |
| n | 12 | 12 | 12 | 12 |
| Age, years | 43.5 (37.75–59.00) | 43.5 (37.75–60.00) | 48.5 (41.0–53.8) | 49.0 (41.0–53.5) |
| Sex, male/female | 9/3 | 3/9 | 9/3 | 3/9 |
| Disease status, active/remission | 12/0 | NA | NA | NA |
| Disease duration, years | 3.5 (1.5–5.0) | NA | NA | NA |
| 5-ASA use, n (%) | 12 (100.0) | 0 (0.0) | 0 (0.0) | 0 (0.0) |
| **Clinical course, n (%)** |  | **NA** | **NA** | **NA** |
| -Chronic relapsing | 8 (66.7) | NA | NA | NA |
| -Initial-onset | 3 (25.0) | NA | NA | NA |
| -Chronic continuous | 1 (8.3) | NA | NA | NA |
| **Disease extent, n (%)** |  | **NA** | **NA** | **NA** |
| -Ileocecal region involvement | 2 (16.7) | NA | NA | NA |
| -Proctitis | 3 (25.0) | NA | NA | NA |
| -Left-sided colitis | 5 (41.7) | NA | NA | NA |
| -Pancolitis | 2 (16.7) | NA | NA | NA |
| **Truelove/Witts classification, n (%)** |  | **NA** | **NA** | **NA** |
| -Mild | 6 (50.0) | NA | NA | NA |
| -Moderate | 2 (16.7) | NA | NA | NA |
| -Severe | 4 (33.3) | NA | NA | NA |
| Extraintestinal manifestations, n (%) | 0 (0.0) | NA | NA | NA |
| Complications, n (%) | 0 (0.0) | NA | NA | NA |
| Corticosteroid use, n (%) | 1 (8.3) | 0 (0.0) | 0 (0.0) | 0 (0.0) |
| Biologic use, n (%) | 0 (0.0) | 0 (0.0) | 0 (0.0) | 0 (0.0) |
| JAK inhibitor use, n (%) | 0 (0.0) | 0 (0.0) | 0 (0.0) | 0 (0.0) |
| Antibiotic use within 3 months, n (%) | 0 (0.0) | 0 (0.0) | 0 (0.0) | 0 (0.0) |
| PPI use, n (%) | 0 (0.0) | 0 (0.0) | 0 (0.0) | 0 (0.0) |
| Prior intestinal surgery, n (%) | 0 (0.0) | 0 (0.0) | 0 (0.0) | 0 (0.0) |

Note: Data are presented as median (interquartile range) or n (%). NA indicates not applicable to non-UC participants. UC, ulcerative colitis; UC_S, healthy spouses of UC patients; HC, healthy controls; HC_S, healthy spouses of healthy controls; 5-ASA, 5-aminosalicylic acid; PPI, proton pump inhibitor.

Supplementary Table S2. Clinical characteristics of individual UC patients

| **Patient ID** | **Sex** | **Age** | **Disease duration** | **Clinical type** | **Disease extent** | **Mayo score** | **Truelove/Witts classification** | **Extraintestinal manifestations** | **Complications** | **Corticosteroid** | **5-ASA** | **Antibiotic** | **Biologic** | **JAK inhibitor** | **PPI** | **Prior intestinal surgery** |
| --- | --- | --- | --- | --- | --- | --- | --- | --- | --- | --- | --- | --- | --- | --- | --- | --- |
| UC01 | F | 58 | 5 years | Chronic relapsing | Ileocecal | 7 | Mild | No | No | No | Mesalazine | No | No | No | No | No |
| UC02 | F | 45 | 8 years | Chronic relapsing | Rectum sigmoid colon | 8 | Moderate | No | No | No | Mesalazine | No | No | No | No | No |
| UC03 | F | 60 | 30 years | Chronic relapsing | Rectum | 12 | Severe | No | No | Yes | Mesalazine | No | No | No | No | No |
| UC04 | M | 42 | 5 years | Chronic relapsing | Left-sided colon | 8 | Severe | No | No | No | Mesalazine | No | No | No | No | No |
| UC05 | M | 59 | 1 month | Initial episode | Ileocecal | 3 | Mild | No | No | No | Mesalazine | No | No | No | No | No |
| UC06 | M | 41 | 2 years | Chronic relapsing | Left-sided colon | 9 | Severe | No | No | No | Mesalazine | No | No | No | No | No |
| UC07 | M | 64 | 3 years | Chronic relapsing | Left-sided colon | 12 | Severe | No | No | No | Mesalazine | No | No | No | No | No |
| UC08 | M | 59 | 2 years | Chronic continuous | Left-sided colon | 3 | Mild | No | No | No | Mesalazine | No | No | No | No | No |
| UC09 | M | 31 | 4 years | Chronic relapsing | Rectum | 3 | Mild | No | No | No | Mesalazine | No | No | No | No | No |
| UC10 | M | 37 | 4 years | Chronic relapsing | Entire colon | 3 | Mild | No | No | No | Mesalazine | No | No | No | No | No |
| UC11 | M | 31 | 1 month | Initial episode | Rectum | 8 | Moderate | No | No | No | Mesalazine | No | No | No | No | No |
| UC12 | M | 38 | 1 month | Initial episode | Entire colon | 3 | Mild | No | No | No | Mesalazine | No | No | No | No | No |
| UC_S01 | M | 65 | NA | NA | NA | NA | NA | NA | NA | No | No | No | No | No | No | No |
| UC_S02 | F | 41 | NA | NA | NA | NA | NA | NA | NA | No | No | No | No | No | No | No |
| UC_S03 | M | 60 | NA | NA | NA | NA | NA | NA | NA | No | No | No | No | No | No | No |
| UC_S04 | M | 46 | NA | NA | NA | NA | NA | NA | NA | No | No | No | No | No | No | No |
| UC_S05 | F | 43 | NA | NA | NA | NA | NA | NA | NA | No | No | No | No | No | No | No |
| UC_S06 | F | 60 | NA | NA | NA | NA | NA | NA | NA | No | No | No | No | No | No | No |
| UC_S07 | F | 38 | NA | NA | NA | NA | NA | NA | NA | No | No | No | No | No | No | No |
| UC_S08 | F | 62 | NA | NA | NA | NA | NA | NA | NA | No | No | No | No | No | No | No |
| UC_S09 | F | 34 | NA | NA | NA | NA | NA | NA | NA | No | No | No | No | No | No | No |
| UC_S10 | F | 37 | NA | NA | NA | NA | NA | NA | NA | No | No | No | No | No | No | No |
| UC_S11 | F | 31 | NA | NA | NA | NA | NA | NA | NA | No | No | No | No | No | No | No |
| UC_S12 | F | 38 | NA | NA | NA | NA | NA | NA | NA | No | No | No | No | No | No | No |
| HC01 | F | 57 | NA | NA | NA | NA | NA | NA | NA | No | No | No | No | No | No | No |
| HC02 | M | 42 | NA | NA | NA | NA | NA | NA | NA | No | No | No | No | No | No | No |
| HC03 | F | 56 | NA | NA | NA | NA | NA | NA | NA | No | No | No | No | No | No | No |
| HC04 | F | 49 | NA | NA | NA | NA | NA | NA | NA | No | No | No | No | No | No | No |
| HC05 | M | 50 | NA | NA | NA | NA | NA | NA | NA | No | No | No | No | No | No | No |
| HC06 | M | 53 | NA | NA | NA | NA | NA | NA | NA | No | No | No | No | No | No | No |
| HC07 | M | 51 | NA | NA | NA | NA | NA | NA | NA | No | No | No | No | No | No | No |
| HC08 | M | 63 | NA | NA | NA | NA | NA | NA | NA | No | No | No | No | No | No | No |
| HC09 | M | 28 | NA | NA | NA | NA | NA | NA | NA | No | No | No | No | No | No | No |
| HC10 | M | 38 | NA | NA | NA | NA | NA | NA | NA | No | No | No | No | No | No | No |
| HC11 | M | 34 | NA | NA | NA | NA | NA | NA | NA | No | No | No | No | No | No | No |
| HC12 | M | 44 | NA | NA | NA | NA | NA | NA | NA | No | No | No | No | No | No | No |
| HC_S1 | M | 58 | NA | NA | NA | NA | NA | NA | NA | No | No | No | No | No | No | No |
| HC_S2 | F | 42 | NA | NA | NA | NA | NA | NA | NA | No | No | No | No | No | No | No |
| HC_S3 | M | 55 | NA | NA | NA | NA | NA | NA | NA | No | No | No | No | No | No | No |
| HC_S4 | M | 50 | NA | NA | NA | NA | NA | NA | NA | No | No | No | No | No | No | No |
| HC_S5 | F | 48 | NA | NA | NA | NA | NA | NA | NA | No | No | No | No | No | No | No |
| HC_S6 | F | 53 | NA | NA | NA | NA | NA | NA | NA | No | No | No | No | No | No | No |
| HC_S7 | F | 50 | NA | NA | NA | NA | NA | NA | NA | No | No | No | No | No | No | No |
| HC_S8 | F | 63 | NA | NA | NA | NA | NA | NA | NA | No | No | No | No | No | No | No |
| HC_S9 | F | 28 | NA | NA | NA | NA | NA | NA | NA | No | No | No | No | No | No | No |
| HC_S10 | F | 38 | NA | NA | NA | NA | NA | NA | NA | No | No | No | No | No | No | No |
| HC_S11 | F | 33 | NA | NA | NA | NA | NA | NA | NA | No | No | No | No | No | No | No |
| HC_S12 | F | 46 | NA | NA | NA | NA | NA | NA | NA | No | No | No | No | No | No | No |

Supplementary Table S3. Reported functions of dominant genera in modules M1–M8

| **Module** | **Dominant genera** | **Reported functions** |
| --- | --- | --- |
| M1 | Streptococcus | Oral–gut axis studies have discussed the potential involvement of oral-associated bacteria, including Streptococcus, in IBD-related intestinal inflammation^[1, 2]^. |
| M2 | Bacteroides | Human gut Bacteroides and Parabacteroides species have broad polysaccharide-utilization capacity^[3]^. |
| M3 | Prevotella | Prevotella has been linked to carbohydrate metabolism and diet-associated gut microbial profiles, but its effects are species-dependent^[4, 5]^. |
| M4 | Prevotella | Prevotella-associated biological effects are heterogeneous and depend on species and ecological context^[4, 5]^. |
| M5 | Clostridium; Eubacterium | Several gut anaerobes, including members of Eubacterium, are associated with butyrate or SCFA production, and SCFAs are involved in epithelial barrier function and immune regulation^[6, 7]^. |
| M6 | Clostridium; Anaerotruncus | Clostridium includes diverse anaerobic gut bacteria with heterogeneous functional potential; direct functional evidence for Anaerotruncus remains limited^[6]^. |
| M7 | Ruminococcus; Roseburia; Eubacterium | Roseburia and some Eubacterium members are commonly discussed as butyrate/SCFA-related gut bacteria involved in intestinal barrier and immune regulation^[6, 7]^. However, R. gnavus has been associated with IBD and inflammatory polysaccharide production^[8, 9]^. |
| M8 | Clostridium; Lachnospiraceae; Coprococcus | Lachnospiraceae includes anaerobic gut bacteria, some of which ferment dietary fiber and produce SCFAs^[6]^. Coprococcus is also discussed among butyrate-producing gut taxa^[6]^. |

1. H. Tanwar, J. M. Gnanasekaran, D. Allison, et al., "Unravelling the Oral-Gut Axis: Interconnection Between Periodontitis and Inflammatory Bowel Disease, Current Challenges, and Future Perspective". *Journal of Crohn's & colitis* 18, no.8 (2024): 1319-1341, <https://doi.org/10.1093/ecco-jcc/jjae028>.

2. A. Wang, Z. Zhai, Y. Ding, J. Wei, Z. Wei,H. Cao, "The oral-gut microbiome axis in inflammatory bowel disease: from inside to insight". *Frontiers in immunology* 15, (2024): 1430001, <https://doi.org/10.3389/fimmu.2024.1430001>.

3. Z. Qu, H. Liu, J. Yang, et al., "Selective utilization of medicinal polysaccharides by human gut Bacteroides and Parabacteroides species". *Nature communications* 16, no.1 (2025): 638, <https://doi.org/10.1038/s41467-025-55845-7>.

4. N. A. Abdelsalam, S. M. Hegazy and R. K. Aziz, "The curious case of Prevotella copri". *Gut microbes* 15, no.2 (2023): 2249152, <https://doi.org/10.1080/19490976.2023.2249152>.

5. P. K. V. Prasoodanan, A. K. Sharma, S. Mahajan, et al., "Western and non-western gut microbiomes reveal new roles of Prevotella in carbohydrate metabolism and mouth-gut axis". *NPJ biofilms and microbiomes* 7, no.1 (2021): 77, <https://doi.org/10.1038/s41522-021-00248-x>.

6. V. Singh, G. Lee, H. Son, et al., "Butyrate producers, "The Sentinel of Gut": Their intestinal significance with and beyond butyrate, and prospective use as microbial therapeutics". *Frontiers in microbiology* 13, (2022): 1103836, <https://doi.org/10.3389/fmicb.2022.1103836>.

7. N. Recharla, R. Geesala and X. Z. Shi, "Gut Microbial Metabolite Butyrate and Its Therapeutic Role in Inflammatory Bowel Disease: A Literature Review". *Nutrients* 15, no.10 (2023): <https://doi.org/10.3390/nu15102275>.

8. M. T. Henke, D. J. Kenny, C. D. Cassilly, H. Vlamakis, R. J. Xavier,J. Clardy, "Ruminococcus gnavus, a member of the human gut microbiome associated with Crohn's disease, produces an inflammatory polysaccharide". *Proceedings of the National Academy of Sciences of the United States of America* 116, no.26 (2019): 12672-12677, <https://doi.org/10.1073/pnas.1904099116>.

9. E. H. Crost, E. Coletto, A. Bell,N. Juge, "Ruminococcus gnavus: friend or foe for human health". *FEMS microbiology reviews* 47, no.2 (2023): <https://doi.org/10.1093/femsre/fuad014>.
